# Imaging moiré flat bands and Wigner molecular crystals in twisted bilayer MoTe_2_

**DOI:** 10.1093/nsr/nwag014

**Published:** 2026-01-20

**Authors:** Yufeng Liu, Yu Gu, Ting Bao, Ning Mao, Shudan Jiang, Liang Liu, Dandan Guan, Yaoyi Li, Hao Zheng, Canhua Liu, Kenji Watanabe, Takashi Taniguchi, Wenhui Duan, Jinfeng Jia, Xiaoxue Liu, Can Li, Yang Zhang, Tingxin Li, Shiyong Wang

**Affiliations:** Tsung-Dao Lee Institute, Shanghai Jiao Tong University, Shanghai 201210, China; Key Laboratory of Artificial Structures and Quantum Control, School of Physics and Astronomy, Shanghai Jiao Tong University, Shanghai 200240, China; Tsung-Dao Lee Institute, Shanghai Jiao Tong University, Shanghai 201210, China; Department of Physics and Astronomy, University of Tennessee, Knoxville, TN 37996, USA; Department of Physics, Tsinghua University, Beijing 100084, China; Max Planck Institute for Chemical Physics of Solids, Dresden 01187, Germany; Tsung-Dao Lee Institute, Shanghai Jiao Tong University, Shanghai 201210, China; Tsung-Dao Lee Institute, Shanghai Jiao Tong University, Shanghai 201210, China; Key Laboratory of Artificial Structures and Quantum Control, School of Physics and Astronomy, Shanghai Jiao Tong University, Shanghai 200240, China; Hefei National Laboratory, Hefei 230088, China; Tsung-Dao Lee Institute, Shanghai Jiao Tong University, Shanghai 201210, China; Key Laboratory of Artificial Structures and Quantum Control, School of Physics and Astronomy, Shanghai Jiao Tong University, Shanghai 200240, China; Hefei National Laboratory, Hefei 230088, China; Tsung-Dao Lee Institute, Shanghai Jiao Tong University, Shanghai 201210, China; Key Laboratory of Artificial Structures and Quantum Control, School of Physics and Astronomy, Shanghai Jiao Tong University, Shanghai 200240, China; Hefei National Laboratory, Hefei 230088, China; Tsung-Dao Lee Institute, Shanghai Jiao Tong University, Shanghai 201210, China; Key Laboratory of Artificial Structures and Quantum Control, School of Physics and Astronomy, Shanghai Jiao Tong University, Shanghai 200240, China; Hefei National Laboratory, Hefei 230088, China; Tsung-Dao Lee Institute, Shanghai Jiao Tong University, Shanghai 201210, China; Key Laboratory of Artificial Structures and Quantum Control, School of Physics and Astronomy, Shanghai Jiao Tong University, Shanghai 200240, China; Hefei National Laboratory, Hefei 230088, China; Research Center for Electronic and Optical Materials, National Institute for Materials Science, Tsukuba 305-0044, Japan; Research Center for Materials Nanoarchitectonics, National Institute for Materials Science, Tsukuba 305-0044, Japan; Department of Physics, Tsinghua University, Beijing 100084, China; Tsung-Dao Lee Institute, Shanghai Jiao Tong University, Shanghai 201210, China; Key Laboratory of Artificial Structures and Quantum Control, School of Physics and Astronomy, Shanghai Jiao Tong University, Shanghai 200240, China; Hefei National Laboratory, Hefei 230088, China; Tsung-Dao Lee Institute, Shanghai Jiao Tong University, Shanghai 201210, China; Key Laboratory of Artificial Structures and Quantum Control, School of Physics and Astronomy, Shanghai Jiao Tong University, Shanghai 200240, China; Hefei National Laboratory, Hefei 230088, China; Tsung-Dao Lee Institute, Shanghai Jiao Tong University, Shanghai 201210, China; Key Laboratory of Artificial Structures and Quantum Control, School of Physics and Astronomy, Shanghai Jiao Tong University, Shanghai 200240, China; Department of Physics and Astronomy, University of Tennessee, Knoxville, TN 37996, USA; Min H. Kao Department of Electrical Engineering and Computer Science, University of Tennessee, Knoxville, TN 37996, USA; Tsung-Dao Lee Institute, Shanghai Jiao Tong University, Shanghai 201210, China; Key Laboratory of Artificial Structures and Quantum Control, School of Physics and Astronomy, Shanghai Jiao Tong University, Shanghai 200240, China; Hefei National Laboratory, Hefei 230088, China; Tsung-Dao Lee Institute, Shanghai Jiao Tong University, Shanghai 201210, China; Key Laboratory of Artificial Structures and Quantum Control, School of Physics and Astronomy, Shanghai Jiao Tong University, Shanghai 200240, China; Hefei National Laboratory, Hefei 230088, China

**Keywords:** twisted bilayer MoTe_2_, moiré flat bands, Wigner molecular crystals, device-scanning tunneling microscopy

## Abstract

Twisted bilayer MoTe_2_ (tMoTe_2_) has recently emerged as an exceptional platform for realizing strongly correlated and topological quantum phases. Yet, its microscopic electronic structure remains largely unexplored. Here, we use scanning tunneling microscopy/spectroscopy (STM/STS) to directly image the moiré flat bands in dual-gated tMoTe_2_ devices with twist angles of 2.3°–3.8°. A dual-gate design allows independent tuning of band filling and displacement field, enabling detailed spectroscopic mapping. We find that the low-energy flat bands are localized at MX and XM sites and form a topological honeycomb lattice at zero electric field. An applied electric field lifts the degeneracy of the layer, driving a transition to two decoupled triangular lattices with trivial topology. Our results match first-principles calculations, revealing K-valley hybridization as the microscopic origin. At large moiré potential, we observe Wigner molecular crystals forming a Kagome lattice at filling *ν*_MX_ = 3, demonstrating electric-field control of topology and correlation in tMoTe_2_.

## Teaser

Scientists demonstrate that electric fields can manipulate moiré flat bands to trigger electrons to crystallize into an ordered Wigner molecular state in twisted bilayer MoTe2.

## INTRODUCTION

Two-dimensional (2D) semiconducting moiré materials have emerged as a highly tunable platform for exploring novel quantum phenomena, such as interaction-driven electronic crystals, correlated topological phases, and unconventional superconductivity [1–18]. Recently, twisted bilayer MoTe_2_ (tMoTe_2_) has attracted significant attention due to the observation of the long-sought fractional quantum anomalous Hall (FQAH) effect [19–34]. Moiré flat bands in semiconducting transition metal dichalcogenide (TMDC) systems can be well described by a 2D electron gas under a modulated moiré potential [35,36]. In TMDC moiré heterobilayers and twisted homobilayers with layer asymmetry, these isolated low-energy flat bands effectively emulate Hubbard physics on a triangular lattice [35,36]. On the other hand, in a band-aligned moiré bilayer, nontrivial band topology can emerge from the hybridization of two sets of moiré flat bands from different layers, forming a honeycomb lattice [5,6]. In real space, the low-energy physics can be understood in terms of carrier hopping on a layer-pseudospin skyrmion lattice, where the layer polarization of the wavefunctions is spatially modulated [5–7]. To date, topological flat bands have been experimentally realized in systems such as WSe_2_/MoTe_2_ heterobilayers [9,10], tMoTe_2_ [19–27], and twisted bilayer WSe_2_ [11,12]. These systems exhibit a wealth of topological quantum phases, such as the integer and fractional quantum anomalous Hall effects.

Recent theoretical work further predicts that intra-moiré interactions can be sufficiently strong to stabilize a Wigner molecular state in TMDC [37–39], leading to a transition from a triangular lattice to an emergent Kagome lattice at a moiré filling factor of *ν* = 3. While TMDC moiré flat bands have been directly visualized in previous STM/STS studies [40–51], direct atomic-scale characterization under *in-situ* tunable displacement fields and filling factors has remained elusive. This gap hinders a unified understanding of the interplay between topology, interactions, and external tuning parameters—which are key to unlocking the full potential of moiré quantum simulators.

Here, we performed STM/STS investigations on tMoTe_2_ with twist angles ranging from 2.3° to 3.8°. Although tMoTe_2_ is the only TMDC material known so far to exhibit FQAH states, STM/STS studies of this system have been scarce due to its instability under ambient conditions. To address this challenge, we employed a monolayer of hexagonal boron nitride (h-BN) as a protective layer, effectively preserving the intrinsic properties of MoTe_2_ while enabling electron tunneling (see Methods). Furthermore, we developed a device structure with two independent back gates, allowing us to directly measure the electronic structure of h-BN–encapsulated tMoTe_2_ under tunable displacement electric fields by fine tuning the gate voltage and the tip bias. Notably, the tunability of the moiré bands through displacement fields provides a critical pathway for unraveling the influence of electron correlations and band topology in this material. Our STS spectra and spatial imaging reveal that the low-energy electron wavefunctions are predominantly localized at XM and MX regions, and form a honeycomb lattice under zero electric field. These spatial distributions are consistent with first-principles calculations, which attribute the K-valley moiré flat band to originating from the hybridization between the top and bottom MoTe_2_ layers. By controlling the moiré potential depth via a perpendicular electric field, we observed Wigner molecular crystals transitioning from a triangular lattice to a Kagome lattice at *ν*_MX_ = 3. These results provide real-space evidence of lattice reconstructions and topological moiré flat bands in tMoTe_2_ and their modulations under displacement electric fields.

## RESULTS AND DISCUSSIONS

### Two setups for the microscopic characterization of tMoTe_2_ moiré superlattice

**Figure 1.**
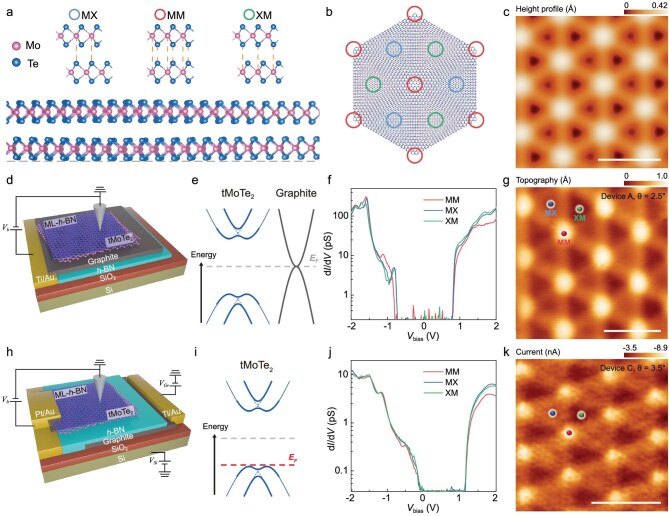
Device structure and lattice relaxation of tMoTe_2_. (a and b) Side and top view of the schematic moiré superlattice of tMoTe_2_. High-symmetry stackings (MX, XM, MM) are highlighted by circles. (c) DFT calculated height profile for 2.65° tMoTe_2_. (d) Schematic of the Setup 1 tMoTe_2_ device for STM measurements. A monolayer of h-BN is used to protect the air-sensitive MoTe_2_ flake while facilitating electron tunneling via the supporting thin graphite film. (e) Schematic of band alignments between tMoTe_2_ and graphite. (f) STS d*I*/d*V* spectra taken at the sites marked in (g). (Setup 1 device A; bias modulation: 20 mV). (g) Experimental STM topography of ∼2.5° tMoTe_2_ (Setup 1 device A: *V*_bias_ = –1.5 V, *I* = 1 nA). (h) Schematic of the Setup 2 tMoTe_2_ device for STM measurements. The silicon gate is used to establish ohmic contact between MoTe_2_ and the electrodes, while the graphite gate is used to tune the filling of tMoTe_2_. (i) Schematic of the filling of tMoTe_2_ bands during measurements. (j) STS d*I*/d*V* spectra taken at the sites marked in (k). (Setup 2 device C; bias modulation: 40 mV). (k) Experimental current topography of ∼3.5° tMoTe_2_ (Setup 2 device C: *V*_bias_ = –0.76 V). Scale bars: 10 nm.

When two layers of MoTe_2_ are twisted by a small angle, a long period moiré superlattice is formed with three high-symmetry stackings, denoted as MM, MX, and XM, as depicted in Fig. 1a and b. Although the effect of period moiré potential can be understood by considering a rigid lattice structure with continuous variation in stacking configurations, the atomically inter- and intra-lattice reconstructions can profoundly influence the moiré band structure. We performed large-scale DFT calculations to address the lattice relaxation effect of tMoTe_2_ with a twist angle of *θ* = 2.65° and 3.89°. The calculation results reveal an out-of-plane corrugation induced by inter-lattice interactions (Fig. 1c), with the MM region elevated by 0.42 Å compared to the XM region, and the XM region is slightly lower by 0.02 Å than the MX region. Additionally, the intralayer strain exhibits a helical chirality, leading to the generation of a substantial pseudomagnetic field. The calculated band structure (including the lattice reconstruction effects) is shown in Fig. S1.

Figure 1d and h illustrates the schematic configurations of the tMoTe_2_ device used for STM measurements. Because undoped-tMoTe_2_ is an insulator—making direct STM measurements unfeasible—we designed two distinct device configurations. In the first setup (Fig. 1d and e), the tMoTe_2_ flake is placed directly on a few-nanometer-thick exfoliated graphite substrate that serves both as the electrode and as the conductive platform for tunneling measurements. This device architecture has been widely employed in previous STM/STS studies of semiconducting TMDCs and their moiré systems. However, in this configuration, the Fermi level of tMoTe_2_ remains locked within the semiconducting gap, pinned by the graphene substrate, which restricts the exploration of its electronic structure under varying filling factors. In the second setup (Fig. 1h and i), a dual back-gating strategy is implemented: the global Si/SiO_2_ gate induces heavy hole doping to establish an ohmic contact between tMoTe_2_ and the platinum electrodes, while the local graphite gate independently controls the Fermi level of tMoTe_2_ under the STM tip. This device structure enables reliable STM/STS measurements of tMoTe_2_ across a range of displacement electric fields and doping densities.

STM measurements were conducted on five tMoTe_2_ devices with slightly different twist angles, ranging from ∼2.3° to 3.8° (Figs S6 and S7). STM imaging reveals a micrometer-size clean area with typical uniaxial strain up to 2% (Fig. S2). As shown in Fig. 1f and j, the STS differential conductance (d*I*/d*V*) spectra reveal the semiconducting gap of tMoTe_2_. Specifically, the valence band (VB) top is located at −0.8 V away from the Fermi level as probed by Setup 1 (Fig. 1f), while another is aligned close to the Fermi level by Setup 2 (Fig. 1j). The measured band gap is ∼1.6 eV. STM topographic imaging reveals structural features that closely resemble the lattice reconstructions predicted by DFT calculations. Notably, the moiré superlattice exhibits XM/MX regions with reduced height, which are separated by domain walls and MM regions (Fig. 1g and k).

### Probing the low-lying moiré flat bands at K and Γ valleys

**Figure 2.**
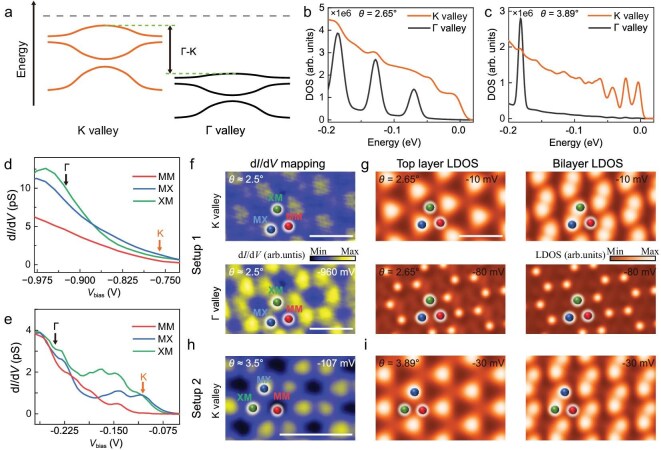
The moiré flat bands from K and Γ valleys of tMoTe_2_. (a) Schematic of band alignments of moiré flat bands from the K and Γ valleys. The moiré flat bands from the K valley lie closer to the Fermi level than those from the Γ valley. (b and c) DFT-calculated DOS spectra of moiré flat bands for 2.65° and 3.89° tMoTe_2_. (d) STS d*I*/d*V* spectra taken at high-symmetry points for a tMoTe_2_ region with θ ≈ 2.5° (Setup 1, Device A), showing weak features from K-valley moiré flat bands. (e) STS d*I*/d*V* spectra taken at a high-symmetry point for a tMoTe_2_ region with θ ≈ 3.5° (Setup 2, Device D), showing clear peak features from K-valley moiré flat bands. The moiré flat bands from the K valley are barely visible in d*I*/d*V* spectroscopy using the Setup 1 device but are significantly enhanced in Setup 2 due to reduced tip-sample separation. (f) Constant-height STS mappings taken at different V_bias_ (Setup 1, Device A; bias modulation: 20 mV). (g) DFT-calculated DOS maps of the top layer and both layers of 2.65° tMoTe_2_ at different energies. (h) Constant-height STS mapping (Setup 2, Device D; bias modulation: 6 mV). (i) DFT-calculated DOS maps of the top layer and both layers of 3.89° tMoTe_2_. Scale bars: 10 nm.

Band folding induced by the periodic moiré potentials creates flat electronic bands in the mini-Brillouin zones. Previous transport and optical measurements suggest that the moiré flat bands at the ±K valleys are closer to the Fermi level than those at the Γ valley [19–22]. To probe the electronic structure and disentangle the contributions from the K and Γ valleys, we performed STS measurements and DFT calculations. As shown in Fig. 2a, the moiré flat bands originating from the K and Γ valleys in tMoTe_2_ are illustrated, with the K-valley bands situated closer to the Fermi level. Figure 2b and c presents the density of states (DOS) spectra for the K and Γ valleys, performed for twist angles of 2.65° and 3.89°. Notably, the energy separation between the K- and Γ-valley bands decreases with decreasing twist angle, highlighting the strong sensitivity of the electronic structure to twist configuration (Fig. S3).

Our calculations reveal that the states at the Γ-valley arise primarily from the d_z²_ orbital of Mo and the *p*_z_ orbital of Te, which exhibit significant out-of-plane orbital character and decay slowly into the vacuum (Fig. S3). In contrast, the K-valley states originate from the in-plane d_xy_/d_x²−y²_ orbitals of Mo and the *p*_x_/*p*_y_ orbitals of Te, exhibiting strong in-plane character and rapid decay outside the MoTe_2_ layer. This distinct decay behavior is directly reflected in our STM measurements under two experimental setups, as the detected STM signal from K-valley states depends critically on tip–sample separations (Fig. S3).

In Setup 1, the VB top is located far from the Fermi level (∼ –0.8 eV), necessitating a relatively large tip–sample distance of ∼1 nm to maintain a stable tunneling condition. This large separation suppresses the weak K-valley features, resulting in only a modest enhancement of the local density of states (LDOS) at MX/XM sites relative to MM sites over a bias range of –0.7 V to –0.9 V (Fig. 2d). In Setup 2, the valence-band top is tuned near the Fermi level using a graphite back gate, allowing STM measurements at a shorter tip-sample distance. This setup enhances the measurement sensitivity of LDOS of the K-valley states. As shown in Fig. 2e, well-resolved LDOS peaks appear at both MX/XM and MM sites. Furthermore, d*I*/d*V* spectra performed at varying tip–sample distances demonstrate that the low-lying K-valley states at MX/XM sites decay much more rapidly with tip retraction compared to the Γ-valley states (Fig. S3). These results confirm that the K-valley moiré bands lie above the Γ-valley bands, establishing that the low-energy physics of tMoTe_2_ is dominated by K-valley states.

Spatially resolved STS measurements further reveal the distinct distributions of K- and Γ-valley states. In Setup 1, two triangular patterns with differing intensities appear at the MX/XM sites at the K-valley band energies, while a honeycomb pattern emerges at the Γ-valley energies. Similar observations are obtained in Setup 2. These spatial patterns are consistent with DFT-calculated DOS maps of the K- and Γ-valley moiré bands (Fig. 2g and i). The key distinction between the valleys lies in their layer-dependent spatial distributions: Γ-valley states form a honeycomb lattice in both layers, whereas K-valley states form a triangular lattice in each layer individually. At zero displacement field, the two triangular lattices overlap to produce a honeycomb pattern; applying a finite displacement field lifts the degeneracy between MX and XM sites, resulting in two independent triangular lattices. In STM measurements under a finite displacement field, this degeneracy lifting manifests as an asymmetry in the observed intensities, with one triangular lattice appearing stronger than the other.

### Modulating K-valley moiré flat bands by displacement electric field

**Figure 3.**
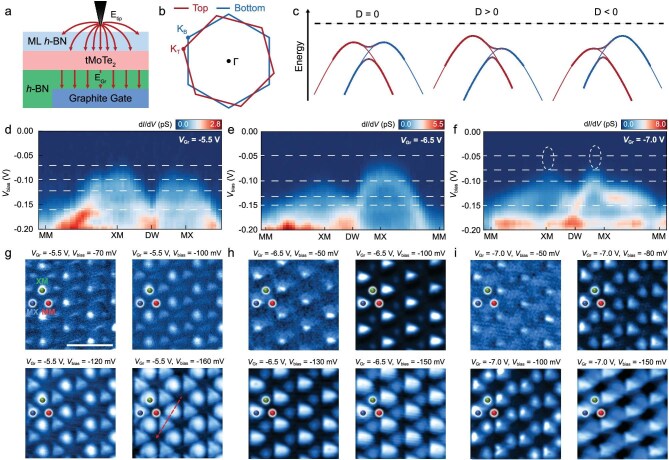
Modulating moiré flat bands from K valleys by displacement electric fields. (a) Schematic of the Setup 2 device. Both the STM tip and the graphite gate introduce displacement fields. (b) Schematic of the Brillouin zone of tMoTe_2_, with regions outlined by red and blue lines representing the top and bottom layers, respectively. (c) Schematic band alignment of moiré flat bands at the K valley under positive/negative displacement fields. In the band structures, the top- and bottom-layer bands are depicted by thin red and blue solid lines. The hybridized moiré flat bands are represented by thick solid lines with a red-to-blue gradient, reflecting interlayer hybridization. At zero field, the top-layer *K*_T_ and bottom-layer *K*_B_ flat bands are degenerate. A displacement field breaks the degeneracy, inducing a relative energy shift and modifying the band structure. (d–f) STS d*I*/d*V* spectra measured along the dashed line in (g) at different graphite gate voltages (Setup 2, Device D), demonstrating the tunability of the moiré flat bands via displacement fields. (g–i) Constant-height current images taken at different *V*_bias_, showing spatial variations in the local density of states (Scale bars: 10 nm). These images highlight the influence of displacement fields on the electronic structure within the XM/MX moiré superlattice. We attribute the intensity at MM sites at *V*_bias_ = –70 mV imaging in (g) to band edge tunneling.

In Setup 2, both the graphite back gate and STM tip contribute to the out-of-plane displacement electric field on tMoTe_2_ (Fig. 3a). We qualitatively studied the electric-field dependence of the K-valley moiré flat bands. Figure 3b illustrates the Brillouin zones of tMoTe_2_, with the regions outlined in red and blue corresponding to the top and bottom layers, respectively. Due to the small twist angle, the Brillouin zones of the two layers are slightly rotated relative to each other in reciprocal space. At zero displacement field, the K-valley flat bands of the top layer (*K*_T_) are degenerate with those of the bottom layer (*K*_B_), as indicated in Fig. 3c. The hybridization of these two sets of moiré flat bands effectively mimics a Kane–Mele model on a honeycomb lattice [52], providing a platform for realizing moiré Chern bands. Applying a displacement electric field lifts the degeneracy between the *K*_T_ and *K*_B_ bands (Fig. 3c). For positive displacement fields, the *K*_T_ bands move closer to the Fermi level while the *K*_B_ bands shift further away (Fig. 3c). Conversely, under negative displacement fields, the *K*_T_ bands move away from the Fermi level and the *K*_B_ bands shift closer (Fig. 3c).

To probe these effects experimentally, we performed dense d*I*/d*V* spectroscopy along the high-symmetry directions while varying the graphite gate voltage to tune the out-of-plane displacement field (Fig. 3d–f and Fig. S4). At –5.5 V gate voltage, the tip-induced and gate-induced displacement fields nearly cancel, resulting in an almost zero net displacement field. Under this condition, the *K*_T_ and *K*_B_ bands are nearly degenerate, forming a honeycomb lattice pattern that persists across different bias voltages (Fig. 3g). Increasing the gate voltage to –6.5 V introduces a positive net displacement field, which shifts the *K*_T_ bands closer to the Fermi level and pushes the *K*_B_ bands further away. This breaks the honeycomb symmetry, with the *K*_T_ bands becoming dominant and forming a triangular lattice at lower biases (Fig. 3h). At even higher displacement fields (e.g. –7.0 V), the *K*_T_ and *K*_B_ bands remain non-degenerate and exhibit triangular lattice patterns at imaging close to the K-valley band edge (Fig. 3i).

We calculated the moiré band structure and DOS spectra using a continuum model incorporating the applied displacement field (Fig. S5). This model captures the key experimental observations: at zero field, the DOS of the K-valley states is degenerate, while increasing the displacement field progressively lifts the degeneracy between MX and XM bands, producing an energy splitting between the corresponding DOS peaks. The magnitude of this splitting grows with increasing electric field, which is in agreement with the experimental results.

### Emergent Wigner molecular crystals in twisted MoTe_2_

Electron density distributions inside the moiré potential can be imaged via tunneling through the valence band edges [51,53]. Using this approach, we examined the electron density distributions at the MX and XM moiré sites under an applied displacement field. Under conditions of strong electron-electron correlations, we observed behavior characteristic of Wigner molecules at the MX moiré sites. The concept of Wigner molecules arises from the interplay of Coulomb repulsion and quantum confinement. Strong electron-electron interactions lead to localized charge distributions, with electrons occupying distinct positions within the confined region.

**Figure 4.**
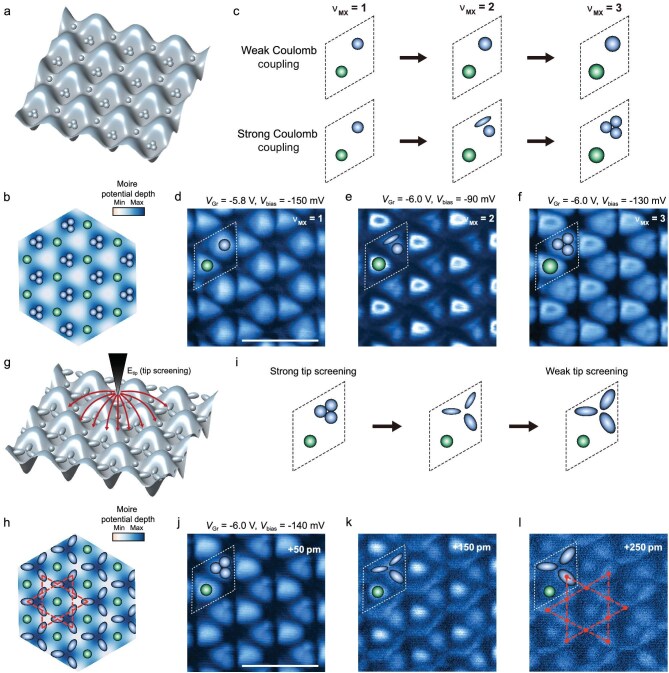
Visualizing and tuning Wigner molecular crystals. (a and b) Schematic of Wigner molecular crystals at a filling of *ν*_MX_ = 3 per moiré site. Under displacement electric fields, the MX moiré potential becomes deeper than the XM moiré, leading to stronger Coulomb interactions. At *ν*_MX_ = 2 and *ν*_MX_ = 3 fillings, electrons spatially separate and localize at distinct positions to minimize mutual repulsion, forming a Wigner molecular crystal. (c) Schematic illustration of electron positions under weak and strong Coulomb interactions. As Coulomb interactions weaken, the Wigner molecule transitions toward a more delocalized state, resembling conventional electronic structures with overlapping wavefunctions. (d–f) Valence-band-edge tunneling maps showing electron densities at different biases and graphite gate voltages. These maps reveal the spatial arrangement of localized electrons and how their distribution evolves under external tuning (Setup 2, Device D). (g) Schematic of the STM tip screening effect, where the local electric field from the tip influences electron localization. (h) Schematic of electron density distribution under weak tip-screening conditions, showing enhanced delocalization of electrons inside MX moiré sites. The red dashed shape outlines the Kagome lattice pattern. (i) Schematic of the reduced tip-screening effect, which enhances electron delocalization. (j–l) Valence-band-edge tunneling maps showing electron densities at different tip-sample separations. The STM tip was gradually retracted from the initial setpoint (Setup 2, Device D, *V*_bias_ = –140 mV, *I* = 18 pA, at XM sites), demonstrating the effect of tip-induced perturbation on electron localization. The red dashed pattern outlines the Kagome lattice in (l). Scale bars: 10 nm.

The Wigner parameter *R*_w_ = *U*/Δ quantifies the competition between Coulomb repulsion energy (*U*) and single-particle energy-level spacing (Δ). In moiré lattices, the displacement field and material properties determine the depth of the moiré potential, setting the energy scale of Δ, while the electron density and dielectric environment control *U*. At small *R_w_*, kinetic energy dominates, and electrons occupy quantum orbitals in a non-interacting sequence, with charge density peaking at the center of the confinement potential (Fig. 4c). As *R_w_* increases, Coulomb interactions become dominant, causing electrons to spatially separate and localize at distinct positions in order to minimize mutual repulsion (Fig. 4a–c). By tuning the displacement field, *R_w_* can be controlled in tMoTe_2_, enabling manipulation of the triangular XM/MX moiré lattices.

Valence-band-edge tunneling maps (Fig. 4d–f) illustrate the evolution of electron configurations in moiré artificial molecules as the electron occupancy per MX site (*ν*_MX_) increases. For *ν*_MX_ = 1, a single charge density node appears at each moiré site center (Fig. 4d), corresponding to a strongly localized electron within the deep moiré potential. At *ν*_MX_ = 2, two distinct charge nodes are observed at MX moiré sites due to Coulomb repulsion exceeding the single-particle energy gap, resulting in spatial separation of the two electrons. When *ν*_MX_ = 3, the charge density evolves into a trimer configuration at MX moiré sites, with three distinct peaks arranged in a triangular pattern. Notably, the charge density exhibits a local minimum at the center of the site, despite the lowest potential being located there (Fig. 4e). This configuration is a direct visualization of Wigner molecule formation, where Coulomb repulsion dominates over the single-particle energy gap.

The dielectric environment plays a critical role in governing Coulomb repulsion between electrons in moiré superlattices. In tMoTe_2_, the effective dielectric constant can be tuned by adjusting the tip-sample distance in the STM junction, which modulates tip-induced screening and, consequently, the strength of Coulomb interactions. This tunability facilitates the exploration of transitions between lattice geometries under different Coulomb interactions. As illustrated in Fig. 4g–i, increasing the tip-sample distance weakens the screening effect, thereby enhancing Coulomb repulsion. This increased repulsion drives greater spatial separation of the trimer electrons, ultimately leading to a nearly perfect Kagome lattice at MX moiré sites, while XM moiré sites retain a triangular lattice configuration (Fig. 4h). These tip-induced behaviors have been confirmed by valence-band-edge tunneling experiments: at short tip-sample distances (Fig. 4j), the trimer remains tightly packed at the moiré center; at medium distances (Fig. 4k), the trimer begins to separate into an irregular three-arm configuration; and at larger distances (Fig. 4i), the trimer achieves further separation, forming a highly ordered Kagome lattice at MX moiré sites.

Wigner molecular crystals are periodic arrangements of Wigner molecules, where localized multi-electron states self-organize into ordered patterns due to the interplay between Coulomb repulsion and the periodic moiré potential. For *ν*_MX_ = 3, electrons form molecular states within each moiré unit cell, with their periodic positioning at MX sites giving rise to a Kagome lattice. These Kagome crystals should exhibit distinct band structures, making them promising platforms for studying correlated quantum phases and exotic electron ordering [37–39].

## CONCLUSIONS AND OUTLOOK

Our study presents a comprehensive atomic-scale investigation of the electronic properties of twisted MoTe_2_, uncovering the intricate interplay between moiré flat bands, the moiré potential, and displacement electric fields. By employing a protective h-BN monolayer and a dual-gating technique, we achieved precise control over the electronic structure, enabling direct observation of low-energy moiré flat bands and the field-induced formation of Wigner molecular crystals. These findings establish a microscopic framework for understanding moiré flat band topology and interaction-driven phases in tMoTe_2_, while demonstrating that displacement fields serve as a powerful tuning knob for engineering emergent quantum phases. Moving forward, minimizing tip-induced effects will be essential in order to further explore and realize novel interaction-driven quantum states.

### Additional notes

During the preparation of this manuscript, we became aware of a recent work that also reports the STM/STS study on tMoTe_2_ [54].

## Supplementary Material

nwag014_Supplemental_File

## Data Availability

All data and code are available from the Corresponding authors upon reasonable request.
