# Supplementary material for: Imaging moiré flat bands and Wigner molecular crystals in twisted bilayer MoTe2
Source: Natl Sci Rev. 2026 Jan 20;13(4):nwag014. doi: 10.1093/nsr/nwag014 (PMC12892358; doi:10.1093/nsr/nwag014)
Supplement: nwag014_Supplemental_File [file nwag014_supplemental_file.pdf]

Supplementary Data for

Imaging moiré flat bands and Wigner molecular crystals in twisted bilayer $MoTe_2$

Yufeng Liu[1,2†], Yu Gu[1†], Ting Bao[3,4†], Ning Mao[5], Shudan Jiang[1], Liang Liu[1,2,8], Dandan Guan[1,2,8], Yaoyi Li[1,2,8], Hao Zheng[1,2,8], Canhua Liu[1,2,8], Kenji Watanabe[6], Takashi Taniguchi[7], Wenhui Duan[4], Jinfeng Jia[1,2,8], Xiaoxue Liu[1,2,8], Can Li[1,2*], Yang Zhang[3,9*], Tingxin Li[1,2,8*], and Shiyong Wang[1,2,8*]

[1]Tsung-Dao Lee Institute, Shanghai Jiao Tong University, Shanghai 201210, China

[2]Key Laboratory of Artificial Structures and Quantum Control (Ministry of Education), School of Physics and Astronomy, Shanghai Jiao Tong University, Shanghai 200240, China

[3]Department of Physics and Astronomy, University of Tennessee, Tennessee 37996, USA

[4]Department of Physics, Tsinghua University, Beijing 10084, China

[5]Max Planck Institute for Chemical Physics of Solids, Dresden 01187, Germany

[6]Research Center for Electronic and Optical Materials, National Institute for Materials Science, Tsukuba 305-0044, Japan

[7]Research Center for Materials Nanoarchitectonics, National Institute for Materials Science, Tsukuba 305-0044, Japan

[8]Hefei National Laboratory, Hefei 230088, China

[9]Min H. Kao Department of Electrical Engineering and Computer Science, University of Tennessee, Tennessee 37996, USA

[†]Equally contributed to this work.

[*]Corresponding authors. E-mails: lic_18@sjtu.edu.cn; yangzhang@utk.edu; txli89@sjtu.edu.cn; shiyong.wang@sjtu.edu.cn

The PDF file includes:

Methods
References
Figs. S1 to S7

Methods

Device fabrications

The tMoTe$_2$ stacks for STM/STS measurements were fabricated using the standard try transfer method [55] (Fig. S1) in a nitrogen-filled glovebox to prevent the degradation of the MoTe$_2$. First, we prepared electrodes (Ti/Au 10 nm/60 nm) on Si/SiO$_2$ substrates using e-beam lithography and e-beam evaporation. Then, monolayer hBN, monolayer MoTe$_2$, graphite (~ 3-5 nm), and hBN (~10-20 nm) were mechanically exfoliated onto Si/SiO$_2$ substrates and identified via optical contrast. A monolayer hBN was initially picked by a polycarbonate (PC) film stamp. This monolayer hBN was then used to picked up half of the monolayer MoTe$_2$ (which was cut into two pieces with an AFM tip before stacking). The remaining MoTe$_2$ was picked up after rotating it by a small angle controlled by a mechanic rotator. After picking up the bottom graphite and bottom hBN layers, the entire stack was released onto the prepatterned Ti/Au electrodes. The monolayer hBN served as the protection layer for tMoTe$_2$. The bottom graphite served as the tunneling electrodes for the STM tip, while the bottom hBN layer screened inhomogeneities generated by substrate surface roughness. The finished stack was dipped in chloroform, acetone and isopropanol for a few minutes, respectively, to remove the majority of the PC film. Finally, any residual contaminants were cleaned by repeated scanning with an atomic force microscope operated in contact mode (Park NX7, setting contact force ~ 300 nN), in order to obtain an atomically-clean surface for STM/STS measurements. Before measurement, these samples were annealed in ultrahigh vacuum at 150 °C overnight.

To protect the air-sensitive tMoTe$_2$ flakes from degradation while maintaining compatibility with STM imaging, we utilize a monolayer of h-BN as a capping layer. The encapsulation with h-BN not only preserves the structural and electronic integrity of the tMoTe$_2$ but also provides an atomically flat and clean surface essential for high-resolution imaging. The detailed fabrication process of these devices is illustrated in Fig. S6-S7. To ensure an atomically clean surface for STM imaging, we adopt a contact-mode atomic force microscopy scanning procedure. This step effectively removes any residual contaminants from the h-BN surface. Post-cleaning, micrometer-sized clean regions suitable for STM studies are observed, as shown in Fig. S2. These setups and preparation techniques enable precise characterization of the electronic structure and moiré patterns in tMoTe$_2$ with high spatial resolution.

Angle inhomogeneity and strain

The local twist angle and strain of tMoTe$_2$ are determined using a uniaxial hetero-strain model [56], which assumes that one layer is uniaxially strained by a percentage ε at an angle θ_s to one of the MoTe$_2$ crystal lattices, while the other layer remains unstrained but with a twist angle θ_T relative to the first. This model provides three degrees of

freedom for the tensile moiré geometry: the moiré twist angle θ_T, the heterostrain magnitude ε, and the angle of uniaxial strain application θ_S. These three variables can be numerically solved by fitting with the experimentally measured three side moiré wavelengths. In this work, the $MoTe_2$ Poisson ratio is estimated to be $\delta = 0.24$ [57]. Additionally, a statistical analysis was conducted on two samples, and the respective twist angle ranges and strain levels are illustrated in Fig. S2.

STM/STS measurements

STS/STM were carried out using a commercial Unisoku Joule-Thomson STM, a commercial Unisoku 1200JT STM system and a commercial CASAcme cryogen free LT SPM under low temperature (4.3K) and ultra-high vacuum conditions (3×10^{-10} mbar). The tungsten tips were calibrated against the surface state of a Cu (111) single crystal or an Ag (111) single crystal. A lock-in amplifier (521 Hz, 3-20 mV modulation) was used to acquire dI/dV spectra. The STM tip was navigated to samples using the capacitance-guiding technique [58]. The STM images were processed with WSxM software.

Theoretical calculation

Our first-principles calculations are based on density functional theory (DFT) as implemented in the open-source package for material explorer (OpenMX) [59] and Vienna *ab* initio Simulation Package (VASP) [60]. For the $tMoTe_2$ with twist angle 2.65° calculated by OpenMX, we adopt the relaxed structure with moiré wavelength 76.23 Å and get the electronic structure under self-consistent criterion of 7×10^{-4} eV. In the DOS and LDOS calculation in $tMoTe_2$, an $81 \times 81 \times 1$ Monkhorst-Pack *k*-point mesh is applied to sample the Brillouin zone (BZ) and the gaussian smearing of energy is chosen to be 0.007 eV. The valley-resolved DOS is generated by considering the summation of projected components of in-plane atomic orbitals as K valley's contribution and summation of out-of-plane ones as Γ valley's contribution. For the bilayer AB-stacking $MoTe_2$ calculated by VASP, we adopt an in-plane lattice constant of 3.52 Å and apply two different van der Waals (vdW) corrections: DFT-D2 and DFT-dDsC. The ionic potential is treated using the projector augmented wave (PAW) method. Exchange-correlation interactions are handled with the Perdew-Burke-Ernzerhof (PBE) functional within the generalized gradient approximation (GGA). We employ a plane-wave basis set truncated at a cutoff energy of 600 eV. Convergence thresholds are meticulously set at 0.001 eV/Å for forces and 10^{-6} eV for total energy. To sample the three-dimensional BZ, we use an $11 \times 11 \times 1$ Monkhorst-Pack k-point mesh.

To mimic the effects of electric field, we employ a continuum model that incorporates first-harmonic, second-harmonic, and displacement field terms to capture the splitting

of MX and XM states. The K valley and K' valley are connected by time-reversal symmetry, making it sufficient to analyze with one valley. Here, we derive the two-band $\boldsymbol{k}\cdot\boldsymbol{p}$ Hamiltonian as:

$$\hat{H}=\begin{bmatrix}-\frac{(k-K_t+eA)^2}{2m^*}+\Delta_t(r)-\frac{\epsilon}{2} & \Delta_T(r)\\ \Delta_T^\dagger(r) & -\frac{(k-K_b-eA)^2}{2m^*}+\Delta_b(r)+\frac{\epsilon}{2}\end{bmatrix}$$

where $\Delta_{t/b}(r)/\Delta_T(r)$ denotes the intralayer/interlayer moiré potential term, and A is the strain-induced gauge field. To mimic the effect of electric field, we introduce the displacement field term ϵ. The intralayer and interlayer moiré potential terms can be obtained after the Fourier transformation:

$$\begin{aligned}\Delta_t(\boldsymbol{r}) &= 2V_1\sum_{i=1,3,5}\cos(\boldsymbol{g}_i^1\cdot\boldsymbol{r}+l\phi_1)+2V_2\sum_{i=1,3,5}\cos(\boldsymbol{g}_i^2\cdot\boldsymbol{r})\\ \Delta_T &= w_1\sum_{i=1,2,3}e^{-i\boldsymbol{q}_i^1\cdot\boldsymbol{r}}+w_2\sum_{i=1,2,3}e^{-i\boldsymbol{q}_i^2\cdot\boldsymbol{r}}\\ A(\boldsymbol{r}) &= A(\boldsymbol{a}_2\sin(\boldsymbol{G}_1\cdot\boldsymbol{r})-\boldsymbol{a}_1\sin(\boldsymbol{G}_3\cdot\boldsymbol{r})-\boldsymbol{a}_3\sin(\boldsymbol{G}_5\cdot\boldsymbol{r}))\end{aligned}$$

Here, $\boldsymbol{G}_{1,3,5}$ and $\boldsymbol{a}_{1,2,3}$ are the moiré reciprocal vectors in reciprocal space and real space. k is the momentum measured from the Γ point of a single-layer $MoTe_2$, K_t/K_b represents the momentum of the top/bottom layer. The terms $\boldsymbol{g}_i^1/\boldsymbol{g}_i^2$ and $\boldsymbol{q}_i^1/\boldsymbol{q}_i^2$ represent the nearest/second-nearest distances of plane wave bases in the same and different layers.

By fitting the density functional theory band structures, we get the following parameter [28]: m^* = 0.62 m_e, V_1 = 10.3 meV, V_2 = 2.9 meV, w_1 = -7.8 meV, w_2 = 6.9 meV, φ_1 = -75°, Φ/Φ_0 = 0.737. Here, m^* is the electron's effective mass. V_1/V_2 and w_1/w_2 are Fourier components of the intralayer and interlayer strength. Φ/Φ_0 represents dimensionless flux, which quantifies the flux in a moiré unit cell in units of the quantum flux. Employing these parameters, we diagonalize the Hamiltonian and calculate the density of states (DOS) according to:

$$D(E)=\frac{N_e}{(2\pi)^2}\sum_n\int_{\text{BZ}}\delta(E-\epsilon_{n,\mathbf{k}})d^2k$$

where N_e represents the band occupancy and $\delta(E-\epsilon_{n,\mathbf{k}})$ is the delta function centered at the energy E. To approximate the delta function, we use a Gaussian broadening:

$$\delta(E-E_n)\approx\frac{1}{\sqrt{2\pi\sigma^2}}\exp(-\frac{(E-E_n)^2}{2\sigma^2})$$

Here, σ is the smearing parameter, and we set to 5 meV during the calculation.

References

55. Wang L, Meric I, Huang PY, et al. One-Dimensional Electrical Contact to a Two-Dimensional Material. *Science* 2013; **342(6158)**: 614–617.
56. Kerelsky A, McGilly LJ, Kennes DM, et al. Maximized electron interactions at the magic angle in twisted bilayer graphene. *Nature* 2019; **572(7767)**: 95–100.
57. Kang J, Tongay S, Zhou J, Li J, Wu J. Band offsets and heterostructures of two-dimensional semiconductors. *Appl Phys Lett* 2013; **102**: 012111.
58. Li G, Luican A, Andrei EY. Self-navigation of a scanning tunneling microscope tip toward a micron-sized graphene sample. *Rev Sci Instrum* 2011; **82**: 073701.
59. Ozaki T. Variationally optimized atomic orbitals for large-scale electronic structures. *Phys Rev B* 2003; **67(15)**: 155108.
60. Kresse G, Furthmüller J. Efficient iterative schemes for ab initio total-energy calculations using a plane-wave basis set. *Phys Rev B* 1996; **54(16)**: 11169–11186.

Supplementary Material Figures

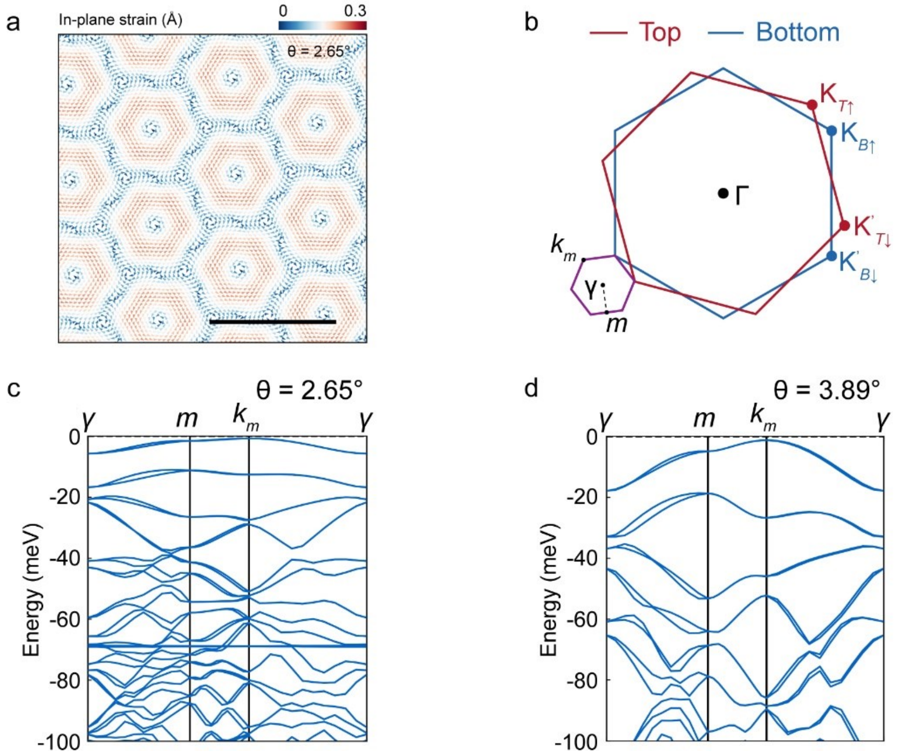

Fig. S1. DFT calculations of $tMoTe_2$ a, Calculated lateral strain distribution in 2.65° $tMoTe_2$. Scale bar represents 10 nm. **b**, Schematic of Brillouin zones: The Brillouin zones of the twisted bilayer $MoTe_2$ (red/blue) and the mini-Brillouin zone of the emergent moiré superlattice (purple). **c,d**, Calculated band structures, incorporating lattice relaxations, for twist angles of 2.65° (c) and 3.89° (d).

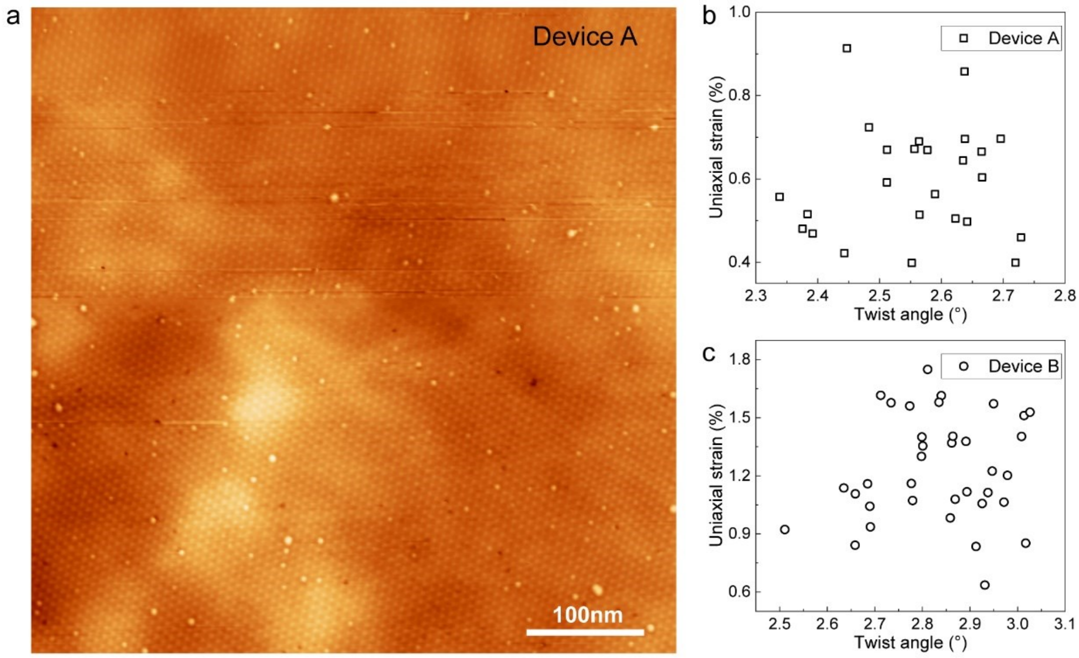

Fig. S2. Large scale STM image of Device A. a, STM topography of $tMoTe_2$ ($\mathbf{V}_{bias}$ = -2.0 V and **I** = 145 pA). The sample is micrometer-size clean with few point defects. **b,c,** The analyzed uniaxial strain as a function of twist angle of device A **(b)** and device B **(c)**.

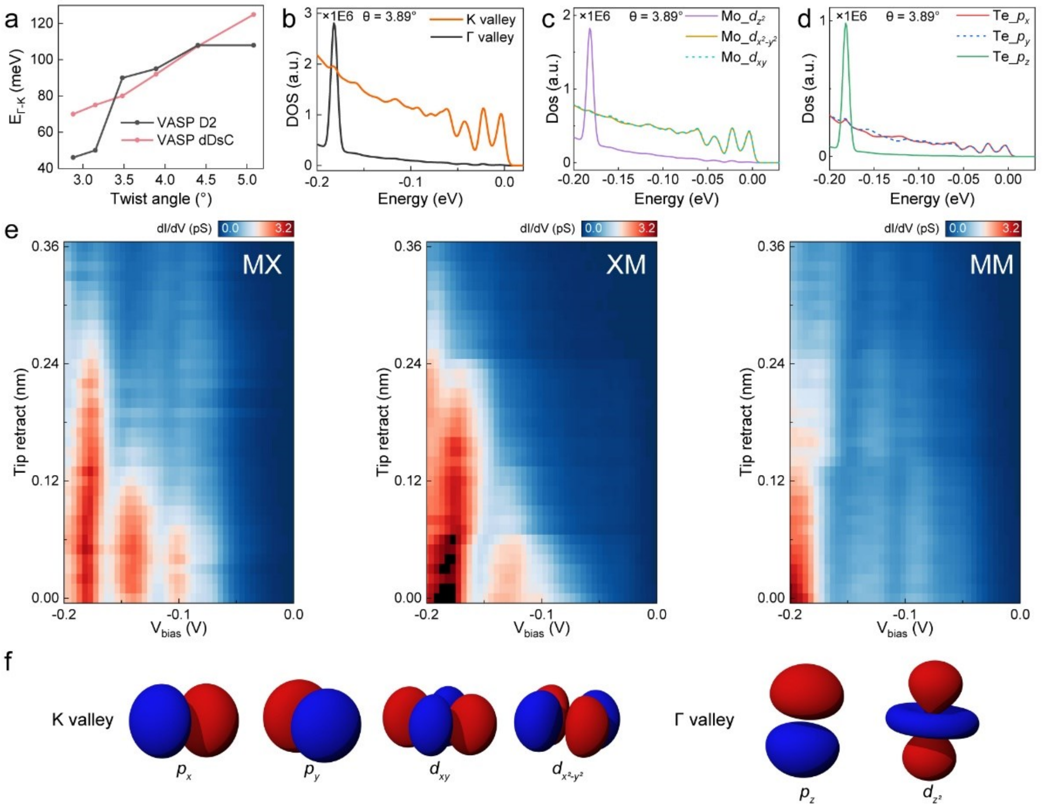

Fig. S3. Orbital characters of Γ and K bands in $tMoTe_2$. a, DFT calculated energy difference between the topmost K-Valley moiré flat band and the topmost Γ-Valley moiré flat band. **b**, DFT-calculated DOS spectra of moiré flat bands for **3.89°** $tMoTe_2$. **c,d**, DFT calculated orbital contributions of Γ and K bands of $tMoTe_2$ with a twist angle of **3.89°**. Γ-valley bands originate from the d_{z^2} orbital of Mo and the p_z orbital Te, which possess out-of-plane orbital characters and therefore decay slowly outside the $MoTe_2$ layer. The K-valley states originate from the in-plane d_{xy} and $d_{x^2-y^2}$ orbitals of Mo and the p_x and p_y orbitals of Te. These wavefunctions have large in-plane orbital characters and decay rapidly outside the $MoTe_2$ layer. **e,** STS dI/dV spectra plots taken at MX, XM, and MM sites with varied tip-sample separations. Bias modulation: 5 mV. (**θ ≈ 3.6°, Setup 2, Device C**). **f**, Cartoons schematically illustrate the orbital composition of states arising from the K- and Γ-valley of the monolayer Brillouin zones.

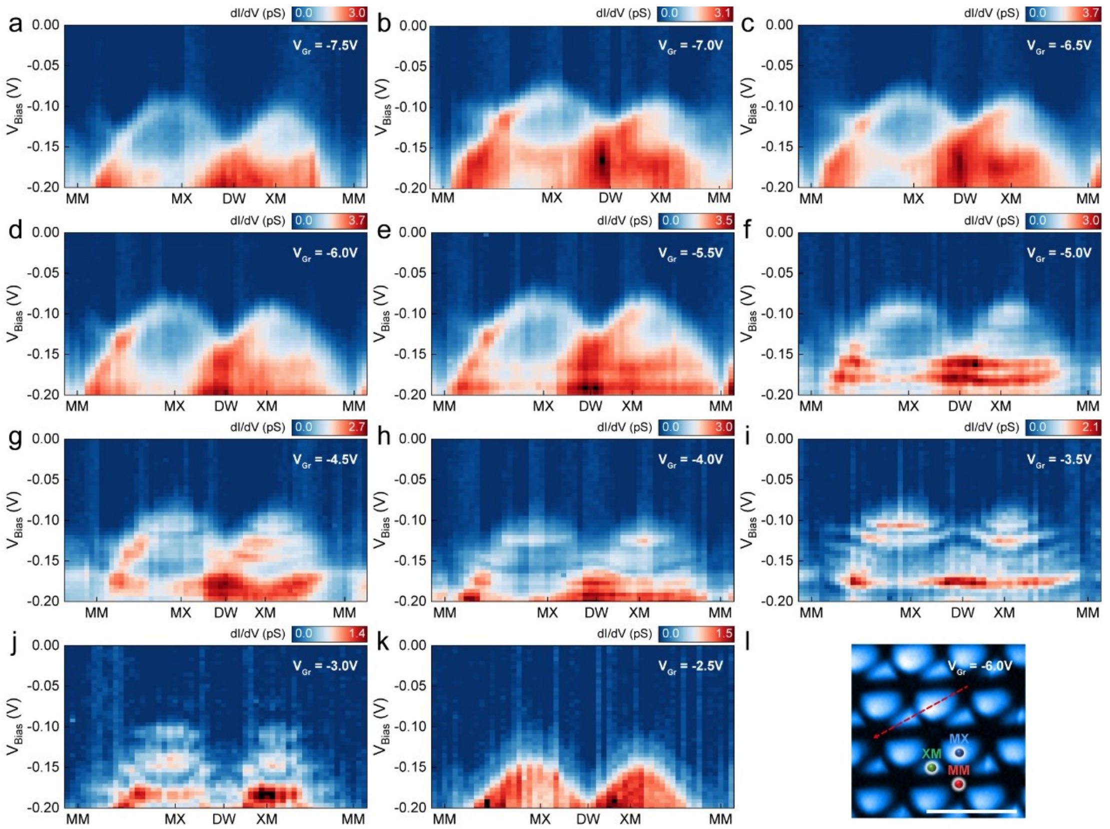

Fig. S4. Modulating moiré flat bands from K valleys by displacement electric fields. **a-k**, STS dI/dV spectra measured along the dashed line in **(l)** at different graphite gate voltages (**θ ≈ 3.3°, Setup 2, Device E**). These spectra demonstrate that the K-valley flat bands are highly tunable via the applied displacement fields. **l**, Constant-height current image revealing the emergence of two distinct triangular lattices at the MX and XM sites, respectively. Scale bars: 10 nm. In the graphite gate range of -2.5 V to -4.0 V, the MX/MX bands are degenerate, and isolated peaks corresponding to different moiré flat bands can be visualized.

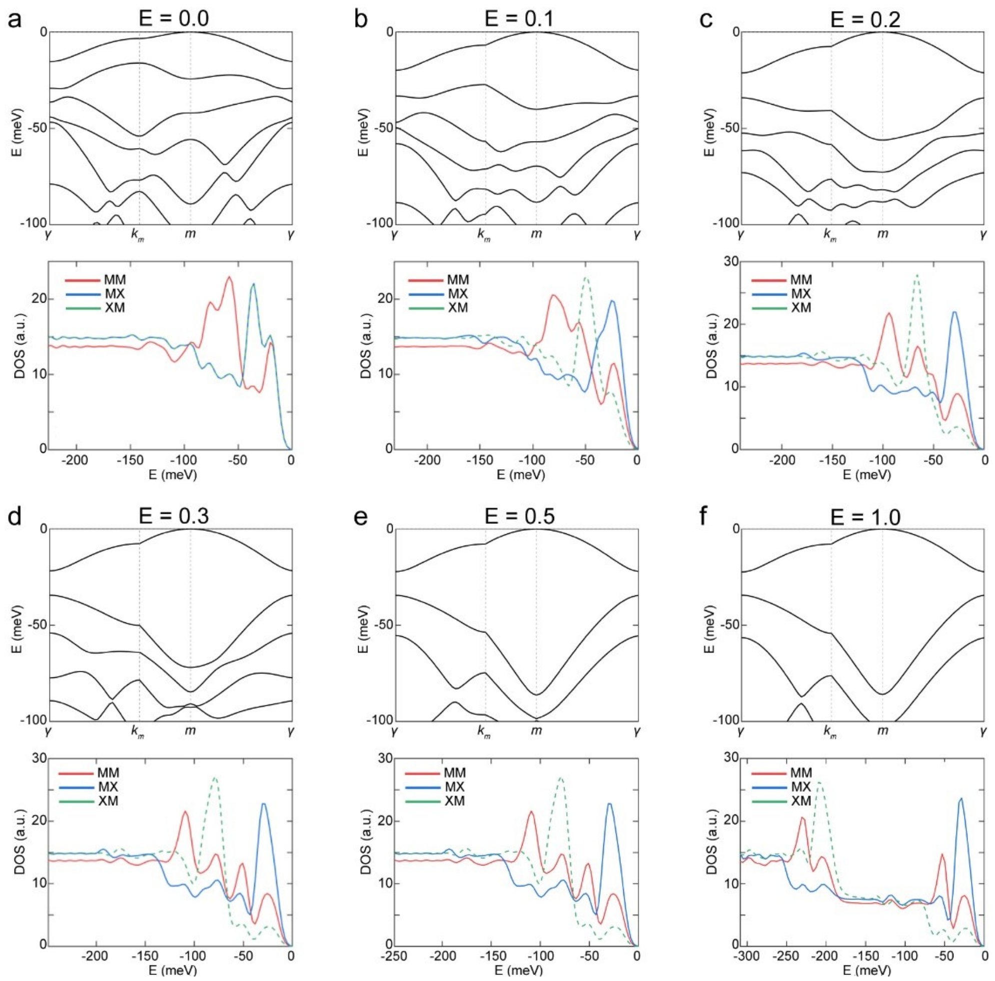

Fig. S5. Continuum Model Calculations of Band Structure and DOS Spectra of 3.65° $tMoTe_2$. The band structure is highly sensitive to displacement electric field, which gradually lifts the degeneracy of the XM/MX states. At zero field, the DOS for the XM and MX states are identical and lie closer to the Fermi level than those of the MM sites. As the displacement field increases, this degeneracy is broken, leading to an energy splitting between the corresponding DOS peaks that grows with the field strength.

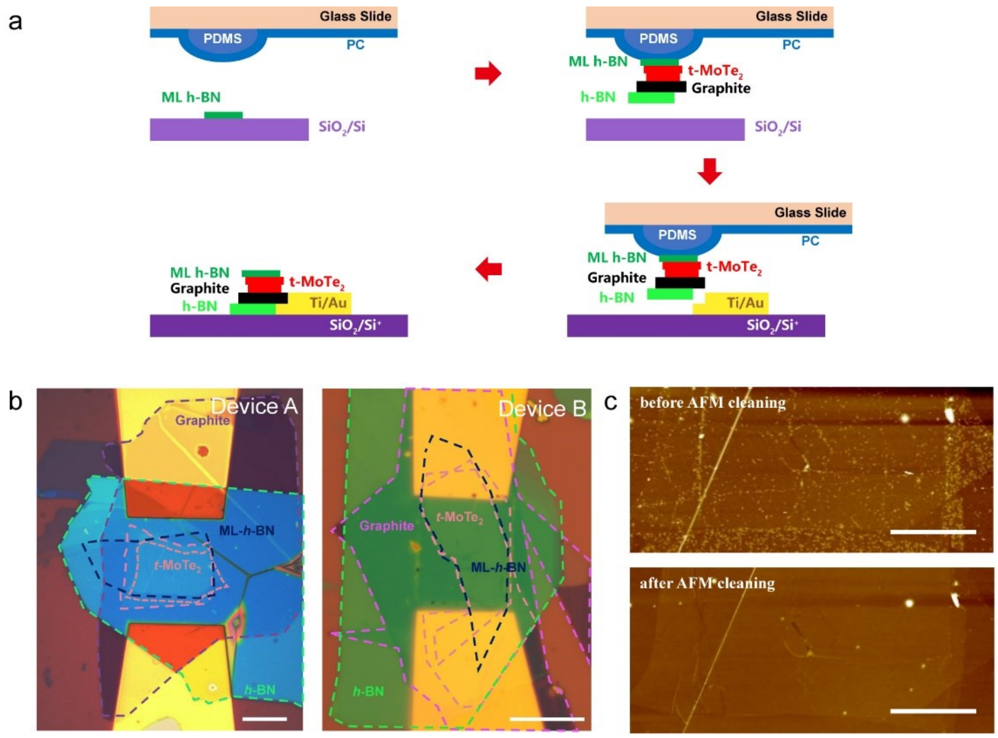

Fig. S6. Device fabrication for Setup 1. a, Schematic of the device fabrication processes. **b,** Optical micrographs of the $tMoTe_2$ device A and device B (from left to right). Dashed lines highlight: pink/blue for $tMoTe_2$/hBN monolayers; purple/green for bottom graphite/hBN flakes. **c,** AFM topography of the $tMoTe_2$ device B before and after contact-mode AFM cleaning. Scale bars: 10 μm (**b**); 5 μm (**c**).

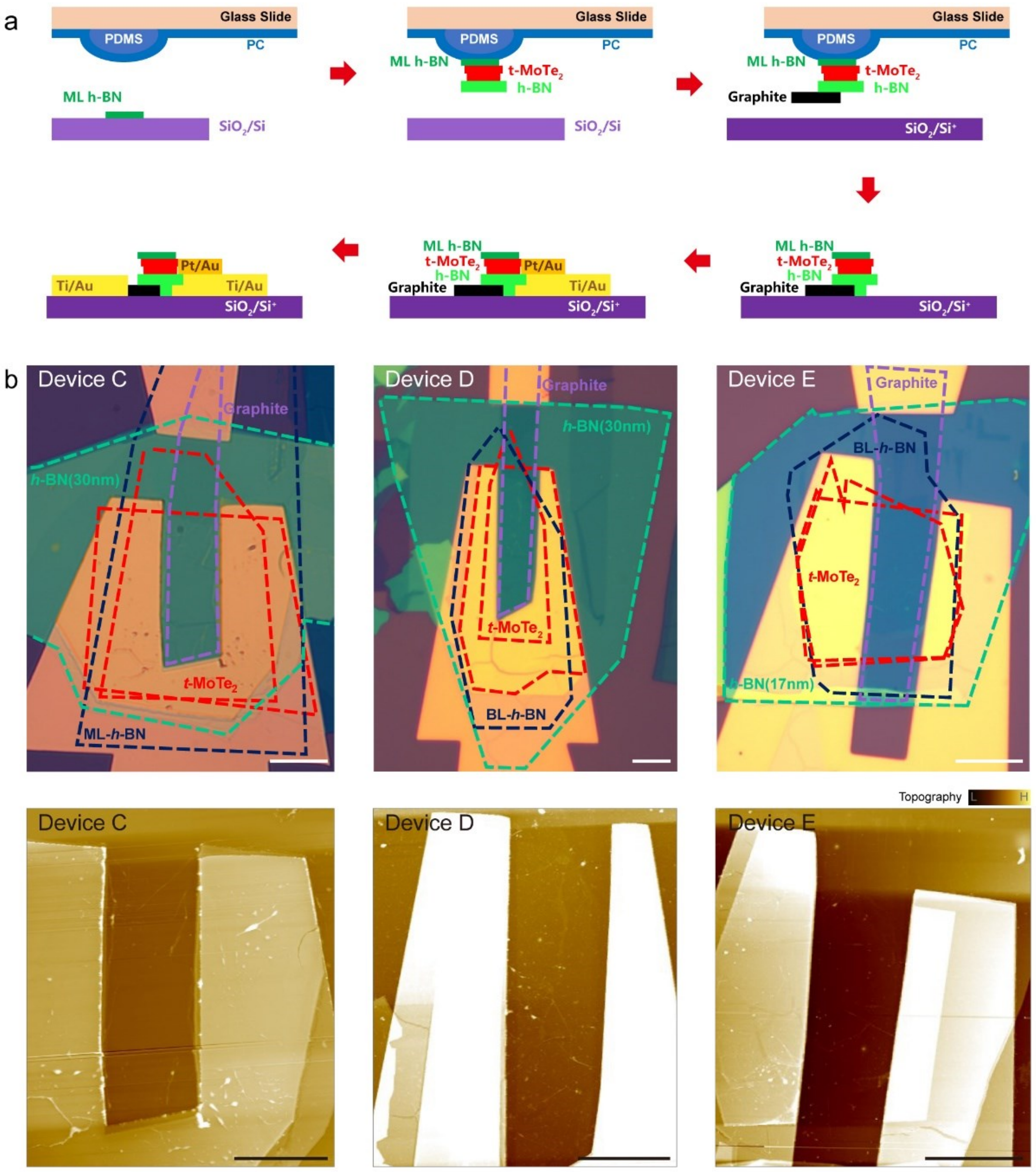

Fig. S7. Device fabrication for Setup 2. a, Schematic of the device fabrication processes. **b,c,** Optical micrographs and AFM images of the $tMoTe_2$ device C, D and E (from left to right). Colored lines highlight: red/blue for $tMoTe_2$/hBN monolayer/bilayer; purple/green for bottom graphite/hBN flakes. Scale bars are 10 μm.
